# Effect of Pharmacist Intervention on a Population in Taiwan with High Healthcare Utilization and Excessive Polypharmacy

**DOI:** 10.3390/ijerph16122208

**Published:** 2019-06-21

**Authors:** Tzu-Chueh Wang, Damien Trezise, Pou-Jen Ku, Hai-Lin Lu, Kung-Chuan Hsu, Po-Cheng Hsu

**Affiliations:** 1 Department of Pharmacy, Chia Nan University of Pharmacy and Science, Tainan City 71710, Taiwan; 2 Department of Applied Foreign Language, Chia Nan University of Pharmacy and Science, Tainan City 71710, Taiwan; damien@mail.cnu.edu.tw; 3 Taiwan Pharmacist Association, Taipei City 10452, Taiwan; kupoujen@yahoo.com.tw; 4 Department of Information Management, Chia Nan University of Pharmacy and Science, Tainan City 71710, Taiwan; hllu2719@gmail.com; 5 Giraffe Pharmacy, Tainan City 71049, Taiwan; gighv206@gmail.com; 6 Yong-xiang Pharmacy, Tainan City 70059, Taiwan; bragihsu19840224@gmail.com

**Keywords:** pharmaceutical care, polypharmacy, drug interactions

## Abstract

Patients with high healthcare utilization are at increased risk of polypharmacy and drug interactions. This study investigated the changes in the number of medications, drug interactions and interaction severity in high frequency outpatients with polypharmacy at hospitals and clinics in Taiwan after home pharmaceutical care, to understand the effectiveness of interventions by pharmacists. This was a retrospective observational study. Cases with excessive polypharmacy (10+ drugs) were selected from the Pharmaceutical Care Practice System database of the Taiwan Pharmacist Association in 2017. After the home care intervention, the number of drug types used decreased 1.89-fold (*p* < 0.001), and the number of medications fell 61.6%. The incidence of drug interaction was 93.82%. In an average case, the incidence of drug interaction after the pharmacist intervention decreased 0.6-fold (*p* < 0.001). The drug most commonly causing interactions was aspirin, followed by diclofenac; also common were three used in diabetes, two psycholeptics and two beta blockers. Among 22 cases of severe drug interaction, seven resulted in increased risk of extrapyramidal symptoms and neuroleptic malignant syndrome. By analyzing the relationship between the side effects of individual drugs and the pharmacokinetic T_max_, a sequential thermal zone model of adverse drug reactions can be established, the value of which could prompt physicians and pharmacists to intervene in order to prevent adverse events. It is concluded that home pharmaceutical care by pharmacists can significantly reduce the number of medications and interactions in patients with excessive polypharmacy and high healthcare utilization.

## 1. Introduction

The National Health Insurance scheme (NHI), introduced in Taiwan in 1995, provides care for all. However, the low copayment, lack of a hierarchical medical and referral system [1], and lack of restrictions on patient visits [2], has resulted in a nearly 17-fold increase in the average number of annual patient visits [3]. Patients with high healthcare utilization not only waste medical resources, but also put themselves at risk of polypharmacy and drug–drug interactions (DDIs) [4,5]. Optimal pharmaceutical care takes place when a pharmacist takes responsibility for the assessment of a condition and the medication prescribed, the development and implementation of therapeutic plans, and the monitoring of treatment efficacy, to ensure that the medications that patients receive are consistent with their indications, are effective, safe, and highly coordinated [6]. In 2007, Article 15 (paragraph eight) of Taiwan’s Pharmacy Act, which concerns functions related to pharmaceutical care, was amended to allow pharmacists to directly provide drug therapy to the general public. In 2010, The Taiwan Medical Association began training pharmacists to implement a home pharmaceutical care plan for sectors of the population with high rates of utilization of healthcare services, enabling pharmacists to provide pharmaceutical care outside of clinical pharmacy services [7].

Home pharmaceutical care in Taiwan is implemented as follows: After cases are selected for this program by the National Health Insurance Administration, the patients continue to attend clinics at their regular medical institutions, but are also visited once a month in their homes by a pharmacist. The aim of these visits is to gain understanding of the patient’s use of medications, and to review the prescriptions given by different medical institutions. If problems are discovered with the medications, the pharmacist will communicate their concerns to the prescribing physician, and the medications will be adjusted accordingly, at the discretion of the physician [8].

Past research on pharmaceutical care in Taiwan has primarily focused on reducing the number of outpatient visits, decreasing the cost of outpatient medical treatment and drugs, decreasing the number of prescriptions written, documenting the cognition and behavior of drug use, and recording service satisfaction. However, such efforts have resulted in little improvement in the number of drug-related problems (DRPs) [3,4,9,10,11,12,13]. In addition, DRP research conducted in other locations may be of little benefit. This is because the characteristics of drug interactions may vary by country, depending on the local conditions, healthcare models, and characteristics of the research community [4,14,15,16,17,18,19,20]. Therefore, the objectives of this study were to investigate the changes in the number of drug types and drug interactions in a population of patients in Taiwan with high healthcare utilization and polypharmacy after home counseling by pharmacists, and to analyze types of drug interactions in order to understand the effectiveness of the pharmacist’s intervention.

## 2. Materials and Methods

High healthcare utilization is defined in Taiwan as more than 90 outpatient visits in the preceding year (excluding visits to dentists, Chinese medicine practitioners, and those associated with rehabilitation) [21]. The inclusion criterion for cases in this study was based on the standards laid out in the National Health Insurance Administration’s 2017 “National Health Insurance Pharmaceutical Care for Patients with High Healthcare Utilization” project [21]. The period before the pharmacist’s intervention was defined as the data collected at the first visit and the period after the intervention was defined as the data collected at the last visit.

This was a retrospective observational study, and was approved by the National Cheng Kung University Hospital Institutional Review Board, which waived the requirement for informed consent (IRB No: B-ER-107-142). The procedure for including cases into the study was as follows: Cases with excessive polypharmacy (more than 10 types of drugs used at the same time [22]) were collected from the Pharmaceutical Care Practice System (Hcare) database of the Taiwan Pharmacist Association for 2017, excluding data entry errors and data collected from single visits. Because there is a strong correspondence between incidence of adverse drug reactions and the use of multiple medications, this study focused on these cases of excessive polypharmacy, with the aim of identifying interactive effects between drugs and offering suggestions for improved practice in the future. The Micromedex interaction query system (IBM Watson Health, Greenwood Village, CO) was used to screen drug interactions; statistical analysis was performed using SPSS (SPSS, Inc., Chicago, IL) and Tableau (Tableau Software, Seattle, WA). We analyzed the differences before and after the pharmacist’s intervention in the number of types of drugs taken, the number of drug interactions, and the severity of drug interactions. The formula for the incidence rate of drug interaction was defined as:

(1) $$\mathrm{incidence}\;\mathrm{rate}=\frac{\mathrm{number}\;\mathrm{of}\;\mathrm{cases}\;\mathrm{with}\;\mathrm{DDIs}}{\mathrm{number}\;\mathrm{of}\;\mathrm{cases}\;\mathrm{included}\;\mathrm{in}\;\mathrm{the}\;\mathrm{analysis}}\times 100\%$$

The classification of DDI severity was based on the Micromedex system and literature review. Changes in the number of medications used, and changes in the number of drug interactions were examined using a paired sample t-test, with the standard of significance set at *p* < 0.05.

We also analyzed the relationships between the side effects of individual drugs and the times to maximum blood concentration (T_max_) in pharmacokinetics in cases randomly chosen from the database. Under the assumption of a single-dose administration, with medications being taken at the same time, and that the drugs did not interfere with each other’s blood concentrations, the side effects of the drugs were positively correlated with the blood concentration, and an additive effect occurred when drugs had similar side effects.

## 3. Results

A total of 469 cases were included in the study. There were 242 male patients (51.6%). The age distribution of cases with potential drug interactions is shown in Table 1. The average age was 70.87 ± 1.03 years and 397 cases (84.7%) were 60 years or older. Among these, 440 cases had drug interactions, for an incidence of drug interactions in patients with polypharmacy and high healthcare utilization of 93.8%.

Before the pharmacist’s intervention, there were 2874 cases of drug interaction, of which only 22 cases (0.8%) were at the contraindicated level; most drug interactions were (95.9%) at the severe or moderate level. The level of evidence was mostly excellent and good which denoted to 40.2% (Table 2).

**Figure 1:**
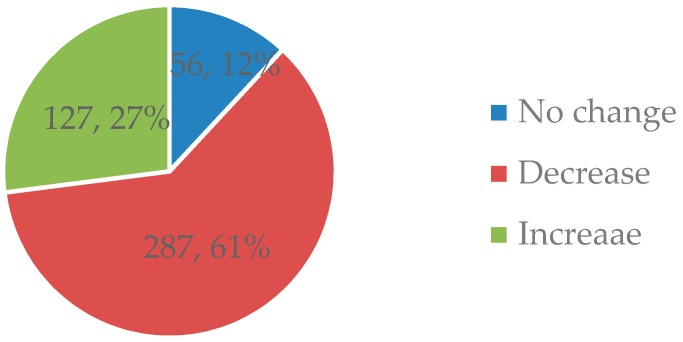
Changes in the number of different types of drugs used after the pharmacist intervention.

The number of drug types used by patients before the intervention (13.01 ± 3.16) was significantly higher than the number used after the intervention (11.12 ± 5.03) (*p* < 0.001). In all, 287 cases took fewer types of drugs after the pharmacist intervention, accounting for 61.6% of the total cases and an average reduction in 1.89 types of drugs used per case (Figure 1).

In addition, the number of drug interactions decreased at all severity levels after the pharmacist intervention. After the intervention, there were 572 fewer drug interactions, a 19.90% reduction. In particular, the level of contraindicated drug interactions decreased 27.27% and severe drug interactions decreased 21.75% (Table 3).

The number of drug interactions decreased after the pharmacist intervention by an average of 0.6 drug interactions per case (before intervention: M = 3.05, SD = 3.00; after intervention: M = 2.45, SD = 2.87, *p* < 0.001). A total of 68.76% of cases had a reduced or unchanged number of drug interactions after the pharmacist intervention (Table 4).

The drug interactions before the pharmacist intervention were further analyzed to select the 10 drugs most involved in drug interactions (Table 5). The results indicated that aspirin with 395 interactions was ranked first, then diclofenac with 298 interactions. Beta blockers, drugs used in diabetes, and psycholeptics were also highly involved in drug interactions.

We then analyzed the types of contraindicated drug interactions before the pharmacist intervention. In the 22 cases at the contraindicated level, 19 cases had evidence at the level of “general”, 17 cases had non-specified onset and 7 cases had a reported result of an increased risk of extrapyramidal symptoms (EPS) and neuroleptic malignant syndrome (NMS), the most commonly-reported result (Table 6).

**Figure 2:**
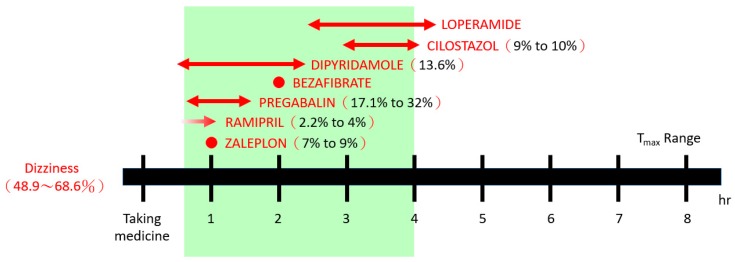
A representative case showing the relationship between dizziness and the time to maximum blood concentration (T_max_) of the individual drugs taken.

We analyzed the relationship between the side effects of individual drugs and the time to maximum blood concentration (T_max_). We found that the time for this case to suffer serious dizziness ranged 0.7–4 hours after administration, and the probability of occurrence was at least 48.9–68.6% (Figure 2).

## 4. Discussion

This study found that the incidence of drug interaction in a population of patients in Taiwan with high healthcare utilization and excessive polypharmacy was more than 90%. This was much higher than that reported in the relevant literature [4,14,15,16,17,18,19,20]. The reason may be that the types of drugs used in a population with high healthcare utilization are complicated, and we deliberately selected cases with excessive polypharmacy in this study; hence, the remarkably high incidence of drug interaction is unsurprising.

Studies show that the probability of developing DRPs increases with the number of drugs prescribed [23]. Based on this result, DRPs could be significantly reduced if the number of drugs used is reduced. In this study, we found that the drug interaction-related DRPs were significantly reduced by the implementation of home pharmaceutical care by pharmacists, which significantly reduced the number of drug types used in cases with high healthcare utilization. This result demonstrated that home pharmaceutical care can improve drug safety, indicating the specific clinical value of pharmacist intervention.

Although the number of medications used by most of the patients decreased after the intervention of the pharmacist, there were still a small number of patients whose number of medications increased. Analysis revealed three major conditions under which this occurred:

(a) The pharmacist recommended medication for an untreated disease.
(b) The patient’s condition had changed and required an adjustment of medication.
(c) The patient was using medications during an acute phase (such as respiratory infections, urinary tract infections, etc.).

In cases with high healthcare utilization, the drugs most commonly causing DDIs were aspirin, diclofenac, drugs used in diabetes, psycholeptics, and beta blocking agents. Among these, aspirin was most likely to interact with metformin and bisoprolol, resulting in a risk of low blood sugar and elevated blood pressure. Diclofenac most often interacted with aspirin and may increase the risk of bleeding. All of these were also among the most used drugs, as reported by the NHI [24]. The best strategy to reduce drug interactions is to use all drug types at the minimum required level [25]. However, the rapidly-aging society of today suffers widely from insomnia, the Three Highs (hypertension, hyperglycemia, hyperlipidemia), degenerative arthritis, and other chronic diseases [26]. In cases where medications are needed to control chronic diseases, medical personnel should exercise caution, particularly pharmacists who are responsible for the regular assessment of a patient’s medications. If the patient has symptoms such as confusion, lethargy, weakness, ambiguity, incontinence, depression or falls, the pharmacist should take the initiative to review all the patient’s medications, identify the drugs that may cause adverse effects, and take the possibility of drug interactions into account [25].

This study showed that seven of the 22 drug interactions in cases with contraindicated severity (the highest level) had increased risk of EPS and NMS. EPS are drug-induced movement disorders in which the pyramidal tract that controls the movement of the nervous system is blocked by drugs that antagonize dopamine D2 receptors and affect the nigrostriatal dopamine pathway, resulting in extrapyramidal side effects. NMS is a rare but potentially fatal complication caused by the use of antipsychotic drugs or other drugs that affect dopaminergic neurotransmission, with clinical symptoms of hyperthermia, muscle rigidity, consciousness disorder, and autonomic instability. The pharmacist should be alert and promptly intervene in cases with such symptoms after the use of drugs that increase the risk of EPS or NMS.

Even after the pharmacists’ interventions, there were still 16 cases of interactive effects at the level of contraindication. The reasons for this may be:

(a) The pharmacist discovered the contraindication and suggested that the prescription be modified, but the physician insisted on maintaining the original prescription.
(b) Although a medication was technically contraindicated, it could actually be used. For example, colchicine is safe for short-term use, but should be discontinued if it reaches toxic concentrations. The pharmacist should pay close attention to the reactions of the case to the medications, and provide counseling on their safe use, in order to prevent adverse reactions, or detect them at an early stage.

These reasons cannot be gleaned simply from inspecting the database. In addition, the purpose of this care program for patients with high healthcare utilization is not primarily to directly solve the drug interaction problem, but to reduce the number of visits these patients pay to hospitals, along with the number of medications that they take, and thus indirectly reduce drug interaction effects.

There are very few studies of the prevalence of different levels of interaction effects. In a 2019 French study of a health insurance database, the prevalence of interaction effects at contraindication levels was found to be 0.2%, which is significantly lower than the rate of 0.76% found in this study. The much higher rate of prevalence in this study may be accounted for by the fact that the sample population consisted of patients with high healthcare utilization of medical services, many of whom used excessive polypharmacy [27]. Patients may use a variety of drugs with similar side effects, which could result in intolerance or serious adverse drug events. By analyzing the relationship between the side effects of individual drugs and the pharmacokinetic T_max_, a sequential thermal zone model of adverse drug reactions can be established, the value of which could prompt physicians and pharmacists to intervene in order to prevent adverse events. Many patients take medications over the long term, increasing their chances of side effects; these chances are again increased as their pill regimen expands. Establishing a polypharmacy risk prediction model could be a possible area of future research.

The potential for drug interactions is based on the pharmacological effects of the drug and may not occur in every patient who uses the drug [25]. Patients with drug interactions may only require close monitoring without any change or adjustment to their dosage. Even in cases in which the potential drug interaction level is higher than severe, clinicians should still weigh the benefits and disadvantages of prescribing. Therefore, pharmacists should seek to comprehend the condition for individual cases and not arbitrarily submit a dear doctor letter based on guidelines alone, so that clinicians and pharmacists can cooperate fully in patient care.

This study had some limitations. Firstly, the population investigated were patients with high healthcare utilization and excessive polypharmacy, and the characteristics of drug interaction in this group may not apply to other groups. Secondly, the lack of certain kinds of information in the Hcare database may also limit the usefulness of this study. In future studies, if more dimensions of data can be collected, such as diagnostic codes and lab data, it may be possible to assess whether or not doctors’ prescriptions are evidence based. Thirdly, we used the Micromedex database and certain drugs may not be included (e.g., traditional Chinese medications), resulting in potential underestimation of the number and severity of drug interactions.

## 5. Conclusions

The NHI has established the NHI PharmaCloud System to improve the safety of medical treatment and drug use for patients and to increase the efficiency of health care resource use. When physicians write prescriptions and pharmacists provide medication adjustments and consultations, this system allows them to access the medical history of patients, which may significantly reduce excessive polypharmacy and cut down on medical expenditures [28,29]. However, can robots and computer programs really replace pharmacists and pharmaceutical care? At least at this stage, it is beyond the power of the NHI PharmaCloud System to reduce drug interactions and DRPs. Furthermore, this study has found that receiving pharmaceutical care from a pharmacist not only provides face-to-face interaction, but also results in a reduction in the average number of medications used per patient and associated interactive effects. This is likely to lead to improved safety in patients’ drug use, and is but one example of the importance of pharmacists in improving health care and reducing morbidity and mortality in at-risk populations.

## Figures and Tables

**Table 1:** Age distribution of cases with potential drug interactions.

Age Group (Years)	Number of Cases (%)
20–39	4 (0.9)
40–59	68 (14.5)
60–79	295 (62.9)
80–99	102 (21.7)
Total	469 (100)

**Table 2:** Analysis of drug interaction before the pharmacist intervention.g

Severity	Level of Evidence			Total (%)
	Excellent	Good	Average	
Contraindicated	2	1	19	22 (0.8)
Severe	94	179	1171	1444 (50.2)
Moderate	148	694	469	1311 (45.6)
Mild	12	26	59	97 (3.4)

**Table 3:** Number of drug interactions before and after the pharmacist intervention.

Severity	Number of Drug Interactions		Percentage Decrease (%)
	Before Intervention	After Intervention	
Contraindicated	22	16	27.3
Severe	1444	1130	21.8
Moderate	1311	1068	18.5
Mild	97	88	9.3
Total	2874	2302	19.9

**Table 4:** Drug interactions reported in the Pharmaceutical Care Practice System database before and after pharmacist intervention on an average per case.

Number of Drug Interactions		Average Count of Drug Interactions	
Number less after intervention	Number greater after intervention	Before	After
647	294	3.05 ± 3.00	2.45 ± 2.87

**Table 5:** The top 10 drugs causing drug interactions.

Name of Drug	Drug Classification	Incidence Number
Aspirin	Antithrombotic Agents	395
Diclofenac	Anti-inflammatory and Antirheumatic Products	298
Bisoprolol	Beta Blocking Agents	242
Propranolol	Beta Blocking Agents	212
Insulin	Drugs Used in Diabetes	157
Tramadol	Analgesics	155
Glimepiride	Drugs Used in Diabetes	152
Zolpidem	Psycholeptics	142
Alprazolam	Psycholeptics	119
Metformin	Drugs Used in Diabetes	118

**Table 6:** The results of contraindicated drug interactions before the pharmacist intervention (*n* = 22).

Drug A	Drug B	Number	Onset	Interaction Results
Aceclofenac	Ketorolac	1	Rapid	Increased gastrointestinal adverse effects
Dicyclomine	Potassium	1	Rapid	Increased risk of gastrointestinal lesions
Oxybutynin	Potassium	1	Rapid	Increased risk of gastrointestinal lesions
Potassium	Tolterodine	1	Rapid	Increased risk of gastrointestinal lesions
Alprazolam	Itraconazole	1	Not Specified	Increased concentration and toxicity of alprazolam
Amisulpride	Chlorpromazine	1	Not Specified	Increased risk of torsades de pointes
Bromocriptine	Sulpiride	1	Not Specified	Reduced efficacy of both
Colchicine	Diltiazem	1	Not Specified	Increased blood concentration and toxicity of colchicine
Dronedarone	Famotidine	1	Not Specified	Increased risk of extended QT-interval
Duloxetine	Rasagiline	1	Not Specified	Caused CNS toxicity or serotonin syndrome
Escitalopram	Rasagiline	1	Not Specified	Increased risk of serotonin syndrome
Levodopa	Sulpiride	1	Not Specified	Reduced efficacy in both
Metoclopramide	Duloxetine	1	Not Specified	Increased risk of extrapyramidal reactions (EPS) and neuroleptic malignant syndrome (NMS)
Metoclopramide	Imipramine	1	Not Specified	Increased risk of EPS and NMS
Metoclopramide	Prochlorperazine	2	Not Specified	Increased risk of EPS and NMS
Metoclopramide	Quetiapine	2	Not Specified	Increased risk of EPS and NMS
Metoclopramide	Sulpiride	3	Not Specified	Increased risk of EPS and NMS
Ropinirole	Sulpiride	1	Not Specified	Reduced efficacy of both
Colchicine	Erythromycin	1	Delayed	Increased blood concentration and toxicity of colchicine
